# Reply to: Accurate population proxies do not exist between 11.7 and 15 ka in North America

**DOI:** 10.1038/s41467-022-32356-3

**Published:** 2022-08-11

**Authors:** Mathew Stewart, W. Christopher Carleton, Huw S. Groucutt

**Affiliations:** 1 Extreme Events Research Group, Max Planck Institutes for Geoanthropology, Chemical Ecology, and Biogeochemistry, Jena, Germany; 2 Department of Classics and Archaeology, University of Malta, Msida, Malta; 3 Department of Archaeology, Max Planck Institute for Geoanthropology, Jena, Germany; 4 Institute of Prehistoric Archaeology, University of Cologne, Cologne, Germany

**Keywords:** Palaeoecology, Palaeontology, Archaeology

**replying to** Pelton et al. *Nature Communications* 10.1038/s41467-022-32355-4 (2022)

In a recent study^1^ we used a novel statistical approach^2^ to investigate whether Late Quaternary changes in North American megafauna populations correlated with changes in human population densities (as is predicted by most “overkill” hypotheses), climate change, or both. Following the design of a recent study by Broughton and Weitzel^3^ and using their datasets, we found no relationship between human and megafauna population levels. We did, however, find a significant positive relationship between megafauna population levels and climate change, suggesting that climate change played a key role in the demise of North America’s megafauna. Pelton et al.^4^ claim that the datasets and analyses used in our study are not “robust enough to support [our] conclusions.” We agree that the North American archaeological and palaeontological records are far from perfect—a point we emphasize in our article—and we welcome the opportunity here to clarify some of the points presented by Pelton and colleagues as well as provide additional analyses that support our original findings.

Pelton and colleagues’ first concern regards the inclusion of potentially non-archaeological dates in the archaeological dataset. Of course, whether a particular age estimate is genuinely associated with human presence at a site or not is often a point of contention among researchers and differing opinions about data validity are to be expected where large, aggregated datasets are concerned. This is particularly the case for the period in question, with debates as to exactly when humans arrived in the Americas having now gone on for decades. Nevertheless, we recognize that uncertainties around which age estimates can be reliably seen as archaeological can have significant impacts on analyses like the ones reported in Broughton and Weitzel^3^ and our own recent study, especially given the already small sample sizes.

With that in mind, we ran two new sets of analyses to see whether more aggressive filtering of the archaeological dataset would produce results that differed from our original findings (see Supplementary Data 1). For the first analysis, we used the vetted dataset of Broughton and Weitzel^3^ which includes only those pre-Clovis period (>13.2 ka) sites that are widely accepted as providing secure evidence for human occupation—Page-Ladson, Meadowcroft Rockshelter, and Paisley Cave. For the second analysis, we removed all dates flagged by Pelton and colleagues as being potentially non-archaeological and restricted our analysis to 14.2–11.7 ka in an effort to avoid “extending human colonization to an increasingly early date.” Despite the more aggressive filtering and tighter chronological constraints in both analyses, all results were consistent with our original findings (Fig. 1). To ensure that the results are comparable between the various studies, we re-calculated the Spearman’s rank correlation coefficients as reported in Broughton and Weitzel using (a) the latest IntCal20 radiocarbon calibration curve and (b) the heavily vetted dataset of Pelton and colleagues. Importantly, the results were essentially the same as those reported by Broughton and Weitzel with only minor differences in the rank-order correlation coefficients (see Supplementary Data 1).

**Fig. 1:**
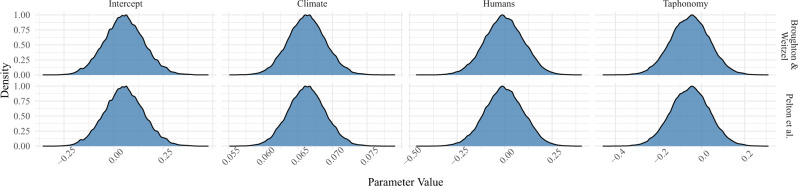
Regression results using vetted archaeological datasets. The effect of climate change, human population size, and taphonomy on megafauna population size using the vetted dates sets of Broughton and Weitzel^3^ (top) and Pelton et al.^4^ (bottom). Note that in both analyses the human posterior estimates overlap zero indicating indicates no relationship between human population size and megafauna population size. On the other hand, the posterior estimates for the climate change parameter do not overlap zero in either analysis indicating a significant effect.

The second potential source of bias identified by Pelton and colleagues concerns the effect of taphonomic decay on the megafauna population proxy. With respect to this potential bias, they raise two points, one relating to the suitability of the proxy we used to control for taphonomic decay, and the other relating specifically to the way in which we used that proxy.

Regarding the first point, Pelton and colleagues argue that the positive correlations we identified between megafauna and climate are the result of autocorrelation. In other words, fossil numbers decrease toward the past as a result of taphonomic decay at the same time that global temperature decreases toward the last glacial maximum, and, therefore, we have not demonstrated a relationship between the two. A simple test of this would be to focus on a period of fluctuating climate, such as the warming of the Bølling-Allerød and the cooling of the Younger-Dryas. Indeed, we present such an analysis above. In this case, despite over half of this time-series encompassing the warming of the Bølling-Allerød moving back in time, the findings are again consistent with our original study.

Pelton and colleagues raise an interesting point, though, about the potential impact of taphonomic decay that we did not address in our original paper. They argue that because of taphonomic decay, time-series regression analyses cannot be used, and instead that it is only possible to identify the point in time during which fossil counts begin to decline toward the present, which they refer to as the “initial decline dates” (IDD). If we assume for a moment they are correct, the IDD itself becomes the keystone piece of evidence for resolving overkill debates. Uncertainty, however, around fossil decay rates leads to significant uncertainty in the changepoint they seek to identify. The key issue, of course, is that the location of the change point will shift along the timeline in accordance with where the taphonomic decay function intersects the true fossil count process (see Fig. 2 for a simple, abstract illustration). Considering that most radiocarbon dates from samples dating to this period have uncertainties that span centuries, and the Clovis period is generally considered to span only a handful of centuries itself, a convincing coincidence between the arrival of Clovis culture and the start of the decline in megafauna may be impossible to defend.

**Fig. 2:**
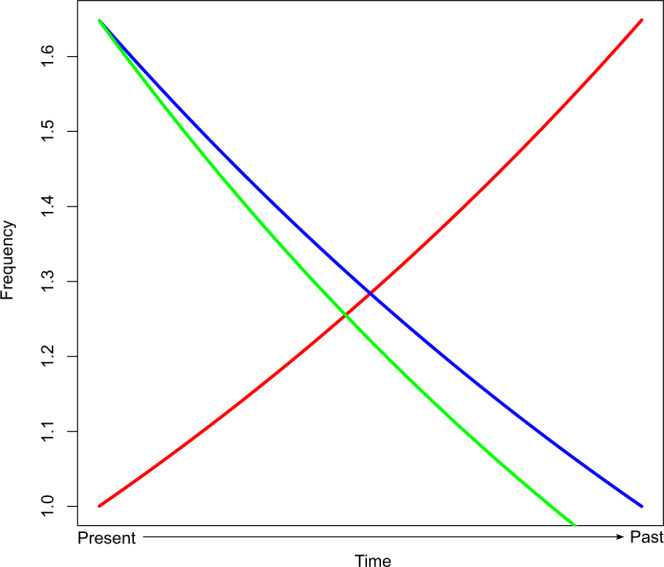
Possible effect of different taphonomic decay functions on change point position. Presented here are two hypothetical taphonomic decay functions (green, blue) and a hypothetical fossil count time-series (red). As shown, a slight shift in the rate of taphonomic decay causes a shift in the time at which it intersects with the fossil curve. Consequently, this will shift the timing of the change point and, given significant uncertainties in radiocarbon dating, may have significant ramifications for identifying coincidences between the appearance of Clovis culture and megafauna declines.

Regarding the second point, Pelton and colleagues imply that we applied an inappropriate mathematical correction to our data resulting in a systematic bias in our megafauna dataset. Surovell et al.’s approach^5^ involves directly altering data with a “correction” equation. This approach is problematic for several reasons. Firstly, it assumes a strictly monotonic taphonomic loss function defined by the exponent in a regression model. It cannot, for example, fluctuate with time-transgressive changes in the taphonomic processes—as might be expected to occur alongside climate change. Secondly, the regression used to estimate the equation’s parameters does not account for chronological uncertainty in the taphonomic proxy data. And finally, the equation is an estimate with its own uncertainties. Using it to alter data directly constitutes a very strong assumption and transfers biases from one regression to any subsequent ones. Importantly, a subsequent analysis would also be unable to re-attribute the variance lost from the correction to another potential explanatory variable (like climate change, or human population pressure). A more standard approach to dealing with confounding variables in regression analyses is to include them as covariates alongside the other variables of interest, which is what we did. Our analysis makes no assumptions about the nature of the taphonomic process and it accounts for chronological uncertainty in the taphonomic proxy data. So, Pelton and colleagues’ suggestion that we applied the wrong correction thereby biasing our results is incorrect—we applied no correction as such.

The third potential source of bias Pelton and colleagues point to concerns the faunal record, and specifically the bias towards heavily dated sites such as Rancho la Brea, Bechan Cave, and Paisley Caves. While it is possible that these sites have impacted our results in some way, this is certainly not the case across all spatial, temporal, and taxonomic scales. For instance, after cleaning Broughton and Weitzel’s dataset to remove dates that possibly derive from the same individual, as well as all dates from disaggregated plant remains from Bechan Cave^1^, the best represented mammoth sites were Boggess Farm and Owl Cave with only three dates each, or 6% of the mammoth dates between 20–10 ka. Likewise, after cleaning, the best represented mastodon sites were Bothwell Farm and Rancho la Brea with only four dates each, or 4% of the mastodon dates between 20–10 ka. Importantly, analysing these cleaned data sets produced results consistent with our initial analysis^1^, suggesting that heavily dated sites were not significantly biasing our results.

In sum, Pelton and colleagues argue that these potential biases systematically affected our results. Here, we have demonstrated that none of these are likely to have significantly influenced our original findings. The only remaining question is whether unaccounted for taphonomic biases in the fauna record ultimately produced our finding of a significant positive relationship between megafauna populations and climate change.

We think that this is unlikely for two key reasons. Firstly, our results were consistent regardless of how the data were treated, which now includes analyses across various temporal, geographic, and taxonomic scales. Importantly, with respects to the former, this now includes analyses of periods dominated by cooling trends (i.e., 20–10 ka) as well as periods dominated by warming trends (i.e., 14.2–11.7 ka), indicating that our findings are not simply the result of unmodelled autocorrelation. Nevertheless, we agree that in future research autocorrelation should be modelled explicitly in order to account for that potential bias in general. Secondly, while imperfect, the taphonomic proxy we used has essentially the same functional form over long periods (exponential-like decline into the past) as the hypothesized taphonomic process across the Americas. So, a strong taphonomic signal in the fossil record would have registered as a positive correlation between fossil counts and the taphonomic proxy over the long interval we analysed even if the rate of decay was not exactly correct. We found no such correlation, implying that a taphonomic process like the one explored by Surovell and Pelton^6^ does not fully account for the patterns in the fossil data over the period of interest at the resolution of our analysis.

Like Pelton and colleagues, we would welcome any new methods or data with the potential to clarify these debates. In the meantime, we propose that available data are analysed in the best available ways. In our paper we acknowledge the limitations of the data we used. Such limitations should also be acknowledged in accounts which make naïve claims for overkill.

In our view, this debate conflates two important points: firstly, what correlations exist within available datasets and, secondly, how reliable the datasets are. For the first point, we have demonstrated a correlation between fluctuations in megafauna numbers and climate change. For the latter, many seem to agree that the available data has serious limitations; that, indeed, is the starting point of our critique of the overkill model. We re-iterate our earlier conclusion that while humans may theoretically have played some kind of indirect role in megafaunal extinctions—different from the widespread overhunting by rapidly expanding human populations typically invoked in overkill hypotheses—if they did, this seemingly occurred within an overarching climatically induced decline in megafaunal populations.

This conclusion only stands, however, given the data we examined. We are aware of at least three ongoing projects aimed at massively increasing the number and quality of reporting of relevant radiocarbon dates ^e.g.^^7^^,^. So, in the near future, it seems likely that these questions about megafauna decline can be revisited, and likely will continue to be revisited as datasets grow. There have also been at least two important relevant methodological developments since our paper was published^8, 9^. The combination of larger datasets and improved methods could change the story once again—a familiar plotline in science.

## Methods

### The data

Three key datasets were used in the present study. The first was compiled by Broughton and Weitzel^3^ and comprised 521 radiocarbon-dated megafauna remains from the US and Canada. In our original study^1^ we conducted some additional cleaning of the megafauna dataset to remove instances where multiple dates might derive from a single individual, as well as dates derived from plant remains. The resulting dataset comprised 432 radiocarbon-dated megafauna remains and was used in the present study. The second dataset was also compiled by Broughton and Weitzel and comprised 938 dates from archaeological contexts obtained from the Canadian Archaeological Radiocarbon Database (CARD). Pelton and colleagues flagged some of the dates as being potentially non-archaeological (see above). Following their recommendations, we cleaned the dataset to remove potentially non-archaeological dates for the present study. The final dataset used was the ~50-year resolved North Greenland Ice Core Project (NGRIP) δ^18^O record. For a full description of the methods and data used in the study we refer readers to Stewart et al.^1^.

### Radiocarbon-dated event count model

For the extended analyses described above, we used the same Radiocarbon-dated Event Count (REC) model approach described in our original article^1^. These are Poisson regression models in which radiocarbon-dated samples comprise count sequences that are then compared to one or more covariates. As described in detail elsewhere^10^, radiocarbon dates contain substantial chronological uncertainty that has to be accounted for in quantitative analyses. REC models attempt to do so by employing a Bayesian hierarchical framework in which probable count sequences produced by randomly sampling individual radiocarbon dates in accordance with their distributions and then binning the dates into a temporal grid. These probable count sequences are treated like samples from a parent population that can be characterised by one or more hyperparameters^2^. The posteriors of the hyperparameters are the main target for estimation and inference, and for present purposes are simply Poisson regression coefficients for three key variables: radiocarbon-dated count sequences of anthropogenic samples (a proxy for human activity); the taphonomic proxy data, which are also radiocarbon-dated count sequences; and probable sequences of oxygen isotopes from NGRIP ice cores that were also sampled in order to account for measurement and chronological uncertainty. After filtering the megafauna database in the ways suggested by Pelton and colleagues and constricting the temporal interval under consideration, we estimated REC model parameters with Markov chain Monte Carlo (MCMC) and then examined the posterior distributions.

### Regression models

In order to make our extended analyses comparable to Broughton and Weitzel^3^, we followed their methods and calculated Spearman rank-order correlation coefficients for the newly filtered and temporally restricted data. The Spearman Rank-order Correlation Coefficient (denoted with the Greek letter ‘rho’) is a non-parametric statistic used to estimate the strength and direction of a monotonic relationship between two variables that can be rank-ordered (i.e., the data are at least ordinal). These correlations were estimated in R using standard built-in functions^11^. Positive values indicate a positive monotonic relationship between the relevant variables, and a negative value indicates an inverse relationship. The statistic ranges from –1 to +1 with the magnitude indicating the strength of the statistical association. We compared the Spearman correlation coefficients given the filtered/temporally-restricted data to those reported by Broughton and Weitzel.

### Reporting summary

Further information on research design is available in the Nature Research Reporting Summary linked to this article.

## Supplementary information

Description of Additional Supplementary Files

Supplementary Data 1

Reporting Summary

## Data Availability

All data require to replicate the analysis are included alongside the published reply article here (Supplementary Data 1).

## Code availability

The code required to replicate the analyses is included alongside the published reply article here (Supplementary Data 1). All analyses were conducted in R (4.03) using a combination of established packages and custom code. The following R packages were used in the analysis and figure production: nimble (0.9.1), ggplot2 (3.3.2), ggpubr (0.4.0), clam (2.3.5), tidyr (1.1.2), and abind (1.4.5).
