# Supplementary material for: Reply to: Accurate population proxies do not exist between 11.7 and 15 ka in North America
Source: Nat Commun. 2022 Aug 11;13:4693. doi: 10.1038/s41467-022-32356-3 (PMC9372036; doi:10.1038/s41467-022-32356-3)
Supplement: Supplementary file 1 — Description of Additional Supplementary Files [file 41467_2022_32356_MOESM1_ESM.pdf]

Supplementary Data 1: Inventory

The Supplementary Data 1 file contains the following:

1) Radiocarbon-dated megafauna dataset (**mfauna.csv**).
2) North Greenland Ice Core Project (NGRIP) $\delta^{18}O$ record (**ngrip.csv**)
3) Calibration radiocarbon database of late Quaternary volcanic eruptions from Byrson, R.A., Ruter, A. (2006. A calibration radiocarbon database of late Quaternary volcanic eruptions. Earth Discusssion 1, 123–124) (**tapho.csv**).
4) Radiocarbon-dated archaeological deposits with potentially non-archaeological dates removed following Pelton and colleagues (**PeltonOK.csv**).
5) R-Markdown file to re-run the Radiocarbon-dated Event Count (REC) models (**REC.Rmd**).
6) R-Markdown file to re-run the Spearman's correlation coefficient analysis (**Spearman.Rmd**).
7) Table presenting the results of the Spearman's correlation coefficient analysis (**Spearman results.docx**).
