# Supplementary material for: Reply to: Accurate population proxies do not exist between 11.7 and 15 ka in North America
Source: Nat Commun. 2022 Aug 11;13:4693. doi: 10.1038/s41467-022-32356-3 (PMC9372036; doi:10.1038/s41467-022-32356-3)
Supplement: Supplementary file 2 — Supplementary Data 1 [file 41467_2022_32356_MOESM2_ESM.zip › Spearman results.docx]

**Supplementary** T**able 1.** **Spearman’s correlation coefficient results.** The rank-order correlation between the megafauna SPDFs and human SPDF between 15–11.7 ka. The first column presents the results of Broughton and Weitzel^3^. The second column is a replication of the first but using the latest radiocarbon calibration curve (IntCal20). The third column is the same as the second but using the vetted dataset of Pelton and colleagues^4^. Note that the results are similar across all analyses, with only minor deviations in the correlation coefficients. Also note that the analyses were conducted using the default settings in R Studio (following Broughton and Weitzel) and so therefore P values were estimated using a t-distribution approximation. The exact P values can be obtained by running the script in Supplementary Data 1.

|  | Broughton and Weitzel^3^ | | Replication | | Pelton et al.^4^ | |
| --- | --- | --- | --- | --- | --- | --- |
|  | rho | *p* value | rho | *p* value | rho | *p* value |
| Horse | -0.43 | <0.0001 | -0.49 | <0.001 | -0.52 | <0.001 |
| Sabertooth | -0.66 | <0.0001 | -0.65 | <0.001 | -0.66 | <0.001 |
| Mammoth | -0.59 | <0.0001 | -0.62 | <0.001 | -0.65 | <0.001 |
| Mastodon | -0.04 | 0.01 | -0.07 | <0.001 | -0.13 | <0.001 |
| Sloth | 0.35 | <0.0001 | 0.30 | <0.001 | 0.23 | <0.001 |
