# Supplementary material for: Reply to: Accurate population proxies do not exist between 11.7 and 15 ka in North America
Source: Nat Commun. 2022 Aug 11;13:4693. doi: 10.1038/s41467-022-32356-3 (PMC9372036; doi:10.1038/s41467-022-32356-3)
Supplement: Supplementary file 3 — Reporting Summary [file 41467_2022_32356_MOESM3_ESM.pdf]

nature portfolio

Corresponding author(s): Mathew Stewart, W. Christopher Carleton, Huw S. Groucutt

Last updated by author(s): 26.07.2022

Reporting Summary

Nature Portfolio wishes to improve the reproducibility of the work that we publish. This form provides structure for consistency and transparency in reporting. For further information on Nature Portfolio policies, see our Editorial Policies and the Editorial Policy Checklist.

Statistics

For all statistical analyses, confirm that the following items are present in the figure legend, table legend, main text, or Methods section.

n/a	Confirmed	
☒	☐	The exact sample size (*n*) for each experimental group/condition, given as a discrete number and unit of measurement
☒	☐	A statement on whether measurements were taken from distinct samples or whether the same sample was measured repeatedly
☐	☒	The statistical test(s) used AND whether they are one- or two-sided<br>*Only common tests should be described solely by name; describe more complex techniques in the Methods section.*
☐	☒	A description of all covariates tested
☒	☐	A description of any assumptions or corrections, such as tests of normality and adjustment for multiple comparisons
☐	☒	A full description of the statistical parameters including central tendency (e.g. means) or other basic estimates (e.g. regression coefficient) AND variation (e.g. standard deviation) or associated estimates of uncertainty (e.g. confidence intervals)
☐	☒	For null hypothesis testing, the test statistic (e.g. *F*, *t*, *r*) with confidence intervals, effect sizes, degrees of freedom and *P* value noted<br>*Give P values as exact values whenever suitable.*
☐	☒	For Bayesian analysis, information on the choice of priors and Markov chain Monte Carlo settings
☒	☐	For hierarchical and complex designs, identification of the appropriate level for tests and full reporting of outcomes
☒	☐	Estimates of effect sizes (e.g. Cohen's *d*, Pearson's *r*), indicating how they were calculated

Our web collection on statistics for biologists contains articles on many of the points above.

Software and code

Policy information about availability of computer code

Data collection	Data were collected with R.4.03 (https://r-project.org)
Data analysis	The code required to replicate the analyses is included alongside the published reply article here (Supplementary Data 1). All analyses were conducted in R (4.03) using a combination of established packages and custom code. The following R packages were used in the analysis and figure production: nimble (0.9.1), ggplot2 (3.3.2), ggpubr (0.4.0), clam (2.3.5), tidyr (1.1.2), and abind (1.4.5).

For manuscripts utilizing custom algorithms or software that are central to the research but not yet described in published literature, software must be made available to editors and reviewers. We strongly encourage code deposition in a community repository (e.g. GitHub). See the Nature Portfolio guidelines for submitting code & software for further information.

Data

Policy information about availability of data

All manuscripts must include a data availability statement. This statement should provide the following information, where applicable:

- Accession codes, unique identifiers, or web links for publicly available datasets
- A description of any restrictions on data availability
- For clinical datasets or third party data, please ensure that the statement adheres to our policy

All data require to replicate the analysis are included alongside the published reply article here (Supplementary Data 1).

Field-specific reporting

Please select the one below that is the best fit for your research. If you are not sure, read the appropriate sections before making your selection.

☐ Life sciences ☐ Behavioural & social sciences ☒ Ecological, evolutionary & environmental sciences

For a reference copy of the document with all sections, see nature.com/documents/nr-reporting-summary-flat.pdf

Ecological, evolutionary & environmental sciences study design

All studies must disclose on these points even when the disclosure is negative.

Study description	In response to Pelton and colleagues, we re-ran our analysis using a more aggressively vetted archaeological dataset. The aims of this re-analysis were to see whether a more aggressively filtered dataset would produce different results to our original findings. The dependent variable in all cases was comprised of radiocarbon-dated events pertaining to megafauna fossils from North America. Two independent variables were used; one comprised archaeological radiocarbon-dated samples from North America; and the other was the North Greenland Ice Core Project (NGRIP) oxygen isotope record.
Research sample	The human radiocarbon sample was obtained from the CARD database (https://www,canadianarchaeology.ca/). The megafauna radiocarbon sample and North Greenland (NGRIP) oxygen isotope record were obtained from published sources.
Sampling strategy	All data used in the present study were obtained from pre-existing databases.
Data collection	All data used in the present study were obtained from pre-existing databases.
Timing and spatial scale	No field or experimental analyses were conducted to obtain the data. All data were collected from pre-existing databases.
Data exclusions	Data were cleaned in two ways and the resulting datasets analyzed separately. For the first analysis, all sites pre-dating 13,200 years BP but excluding Page-Ladson, Meadowcroft Rockshelter, and Paisley Cave were removed. For the second analysis, all sites flagged as non-archaeological by Pelton and colleagues were removed.
Reproducibility	All data and code required to replicate the analysis are provided with the paper.
Randomization	.All available human and megafauna dates were used in the analysis
Blinding	No experiments were conducted for the present study.

Did the study involve field work? ☐ Yes ☒ No

Reporting for specific materials, systems and methods

We require information from authors about some types of materials, experimental systems and methods used in many studies. Here, indicate whether each material, system or method listed is relevant to your study. If you are not sure if a list item applies to your research, read the appropriate section before selecting a response.

Materials & experimental systems

n/a	Involved in the study	
☒	☐	Antibodies
☒	☐	Eukaryotic cell lines
☒	☐	Palaeontology and archaeology
☒	☐	Animals and other organisms
☒	☐	Human research participants
☒	☐	Clinical data
☒	☐	Dual use research of concern

Methods

n/a	Involved in the study	
☒	☐	ChIP-seq
☒	☐	Flow cytometry
☒	☐	MRI-based neuroimaging
